# Growing vegetables in a warming world - a review of crop response to drought stress, and strategies to mitigate adverse effects in vegetable production

**DOI:** 10.3389/fpls.2025.1561100

**Published:** 2025-04-04

**Authors:** Jongwon Park, Se-Hyoung Lee, Joowon Lee, Seung Hwan Wi, Tae Cheol Seo, Ji Hye Moon, Seonghoe Jang

**Affiliations:** ^1^ World Vegetable Center Korea Office, Wanju-gun, Republic of Korea; ^2^ Vegetable Research Division, National Institute of Horticultural and Herbal Science, Rural Development Administration, Wanju-gun, Republic of Korea

**Keywords:** climate change, drought resistance, drought stress, plant responses, vegetable production

## Abstract

Drought stress caused by climate change is increasingly affecting the productivity and quality of vegetable crops worldwide. This review comprehensively analyzes the physiological, biochemical, and molecular mechanisms that vegetable crops employ to cope with drought stress. In particular, it highlights the significance of key hormonal regulation pathways, such as abscisic acid (ABA), jasmonic acid (JA), and ethylene (ET), which play crucial roles in mediating stress responses. Additionally, the role of antioxidant defense systems in mitigating oxidative damage caused by reactive oxygen species (ROS) is discussed. Advances in agricultural technologies, such as the use of smart irrigation systems and biostimulants, have shown promising results in enhancing drought resistance and optimizing crop yields. Integrating these strategies with the development of drought resistant varieties through gene editing and traditional breeding techniques will ensure sustainable agricultural production in drought stressed environments. This review aims to support future research into sustainable agricultural development to enhance drought tolerance in vegetable production and secure global food supply.

## 1 Introduction

Drought stress is one of the environmental factors that most severely affects agricultural productivity worldwide (Yang et al., 2019; Kim et al., 2020; Hanlon et al., 2021). As the frequency and intensity of droughts increase due to climate change, understanding and enhancing drought resistance of vegetable crops has become even more important. Most vegetable crops are primarily composed of water, making them particularly vulnerable to drought stress that affects their growth and development, and water deficits during critical growth stages result in significant reductions in yield and quality (Liang et al., 2020; Ackah and Kotei, 2021; Mukherjee et al., 2023; Jang et al., 2024). Vegetables are an essential food resource for humans, playing a key role in providing nutrients, vitamins, dietary fiber, antioxidants, and other beneficial phytochemicals. However, despite the increase in global production, the supply remains insufficient (Costlow et al., 2025).

Drought stress induces osmotic and oxidative stress in crops, leading to damage in cellular organelles caused by reactive oxygen species (ROS) (Laxa et al., 2019; Chen et al., 2022). In response to drought stress, vegetable crops engage complex mechanisms, including the hormonal regulation of key gene expression/signaling pathways, the activation of antioxidant enzymes, and protein synthesis and degradation processes (Iqbal et al., 2019; Zhou et al., 2019a; Shin et al., 2021).

Recent research has focused on improving drought resistance by integrating new plant breeding technologies (NPBTs) with traditional breeding methods to develop resistant varieties, providing a major boost to food security and sustainable agricultural development (Shao et al., 2018; Qaim, 2020). In particular, developing genetically modified organisms (GMOs) and gene-edited crops opens new possibilities in this field, playing a vital role in addressing the decline in agricultural productivity caused by climate change (Qaim, 2020).

In addition to advancements in breeding technologies, strategies such as climate-smart agricultural systems and biostimulant applications have shown promise in mitigating the adverse effects of drought on vegetable crops (Kıran et al., 2019; Savarese et al., 2022). In particular, the integration of smart irrigation systems and precision watering techniques, such as drip irrigation, can improve water use efficiency, enhance antioxidant defense mechanisms, optimize osmotic regulation, and effectively modulate key stress-responsive pathways, thereby not only enhancing drought tolerance but also playing a critical role in maintaining sustainable crop productivity and quality (Abdelraouf et al., 2020; Bwambale et al., 2022; Debebe et al., 2025).

This review aims to provide a comprehensive understanding of the physiological, biochemical and molecular responses of vegetable crops to drought stress, while analyzing the effects on vegetable crops from various perspectives. Additionally, through case studies of major vegetable crops, the review analyzes actual research outcomes applied in practice in terms of how much they enhance the drought resistance of vegetable crops and support effective agricultural production strategies.

## 2 Climate change and drought stress: impacts on vegetable crops

Climate conditions significantly influence the yield and quality of vegetable crops. Recently, abnormal weather patterns due to global warming have become more frequent, and global warming due to the increase in atmospheric carbon dioxide is expected to further alter precipitation patterns and distribution (Yang et al., 2019). Typically, drought stress results from insufficient rainfall, but it can also be triggered by prolonged high temperatures, intense sunlight, and soil drying caused by dry winds (Cohen et al., 2021).

Developing countries are facing severe food security crises, partly due to declining annual rainfall, which leads to reduced agricultural productivity (Warner and Afifi, 2014; Change and FAO, 2016; Dinko and Bahati, 2023). Impacts are also seen in temperate zones such as the UK, where changes in rainfall patterns due to climate change are also expected to increase the frequency and intensity of droughts (Hanlon et al., 2021). Additionally, traditional agricultural regions are moving because of more frequent droughts and water shortages (Kanyama Phiiri et al., 2016). In Korea, climate simulation studies predict significant changes in rainfall from September to November between 2030 and 2050, which will impact Chinese cabbage production (Kim et al., 2020).

Drought stress refers to the physiological and biochemical responses of plants when they experience water deficits. It occurs mostly from reduced rainfall that leads to insufficient soil moisture, severely affecting crop growth, development, productivity, and quality. For example, with Chinese cabbage (*Brassica rapa* subsp. *pekinensis*), insufficient water supply during the early heading stage after sowing, leads to retarded leaf development and significantly reduced growth rates. Leaves also become smaller in size and number, and tend to yellow, wilt or become necrotic (Jang et al., 2024). Similarly, tomato (*Solanum lycopersicum* L.) requires adequate water supply, especially during early growth and fruit formation (Mukherjee et al., 2023). In addition, water availability is essential to the growth of pepper (*Capsicum annuum*), with higher water requirement during flowering and fruit-set (Ichwan et al., 2022). The duration, intensity, and frequency of drought stress also affect the growth and productivity of crops such as cabbage (*Brassica oleracea* var. *capitata*), that under prolonged drought stress causes decreased leaf size, resulting in reduced photosynthesis and a significant reduction in final yield (Ackah and Kotei, 2021).

Climatic conditions affect the yield of various vegetables, with insufficient water supply inhibiting the growth and development of plant organs such as leaves, stems and roots, negatively impacting overall plant growth. Thus, to maintain agricultural sustainability under abnormal weather patterns including drought caused by climate change, it is imperative to further research drought resistance in plants; including a deeper understanding molecular/physiological mechanisms in response to drought stress, to the development of optimized agricultural practice and breeding of drought tolerant crops.

## 3 Physiological response mechanisms

Vegetable crops exhibit physiological changes in response to environmental stimuli, such as drought stress, which significantly impact crop quality and yield (Ullah et al., 2019; Kapoor et al., 2020; Qiao et al., 2024). Plants lose more water through transpiration from their leaves than they can take in through their roots when soil moisture levels drop as a result of less rainfall, heat, sunlight, or dry winds (Kirschbaum and McMillan, 2018). In response, plants expand their root systems to absorb more water and minimize water loss by closing stomata on their leaves (Fahad et al., 2017). Understanding the mechanisms behind root expansion, including increasing root length and growing deeper into the soil is critical for developing agricultural strategies to enhance crop productivity. By optimizing water absorption and minimizing water loss, plants can better adapt to drought stress (Tron et al., 2015).

**Figure 1:**
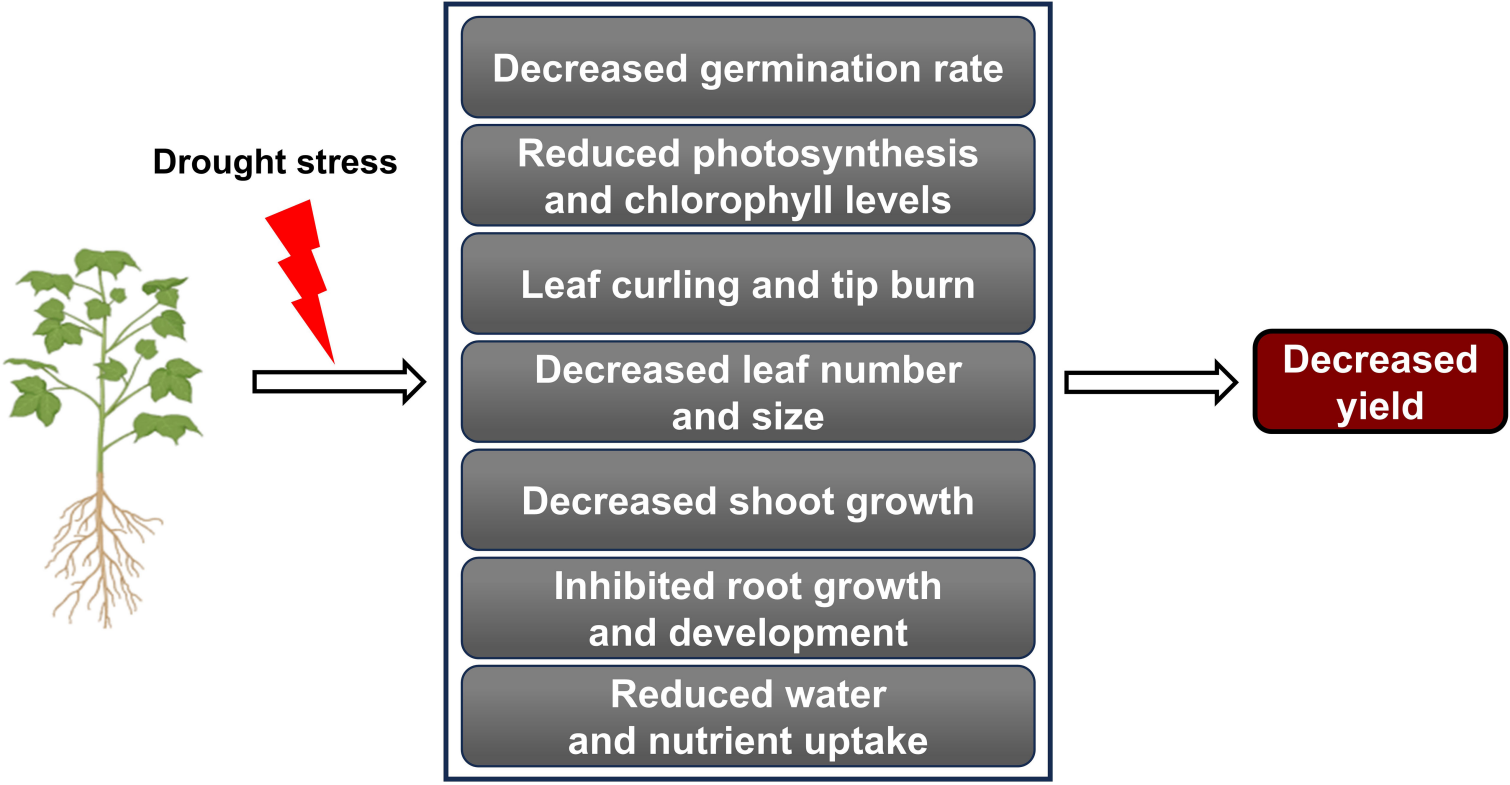
Drought stress impact on plant growth and yield. Drought stress negatively affects various physiological and developmental processes in plants. Various adverse effects caused by drought stress collectively lead to a decline in agricultural productivity.

Under drought stress, plants commonly exhibit leaf changes, including reduced growth, curling, yellowing, tip burn, and permanent wilting (Jang et al., 2024). Stomatal closure is a defense mechanism that plants use to minimize water loss in drought conditions, but it can also reduce photosynthetic efficiency (Pamungkas and Farid, 2022). Research on tomatoes (*Solanum lycopersicum* L.) demonstrated that plants close their stomata to conserve water, but this also lowers internal carbon dioxide levels, leading to reduced photosynthesis under drought stress (Liang et al., 2020). As photosynthesis declines, energy and carbohydrate production needed for growth decrease, ultimately inhibiting overall plant development (Liu et al., 2023b) (**Figure 1**).

In addition, plants maintain cellular ion concentration and osmotic balance through a process called osmoregulation, enhancing their physiological adaptability and increasing the chances of survival under stress (Ozturk et al., 2021). Thus, osmoregulation serves as an important defense mechanism; however, its effectiveness decreases under prolonged stress conditions, as observed in Welsh onion (*Allium fistulosum*), where extended drought stress (28 days) led to a decrease in osmolyte accumulation and an inability to restore physiological homeostasis, ultimately reducing plant survival and productivity (Liu et al., 2023c). Drought stress-induced physiological changes in plants directly affect agricultural productivity, making it increasingly difficult to cultivate vegetable crops in water-scarce regions (Qiao et al., 2024).

## 4 Biochemical response mechanisms

### 4.1 Antioxidant defense system

Drought stress leads to an overproduction of ROS in plant cells, causing oxidative stress that significantly affects physiological functions. ROS, such as hydrogen peroxide (H_2_O_2_), superoxide ion (O_2_^-^), hydroxyl radical (OH^-^), and singlet oxygen can cause detrimental effects on essential cellular components like membranes, proteins, and nucleic acids (Zhou et al., 2019a). To counteract this, plants activate their biochemical defense mechanisms through antioxidant enzymes (Laxa et al., 2019). Superoxide dismutase (SOD) converts superoxide ions into H_2_O_2_ and oxygen (O_2_), which are then further broken down into H_2_O by other antioxidant enzymes. Peroxidase (POD) is another key antioxidant enzyme whose activity increases in response to various abiotic stresses, playing a critical role in scavenging ROS and protecting cells. POD also contributes to lignin biosynthesis, which helps maintain the cell wall and reinforces plant structure (Yang et al., 2022b). Ascorbate peroxidase (APX) also plays a significant role in removing ROS; APX uses ascorbic acid as a substrate to reduce hydrogen peroxide to water, generating dehydroascorbic acid (DHA). DHA can be recycled back into ascorbic acid, allowing continuous antioxidant action (Fujita and Hasanuzzaman, 2022).

Together with SOD, POD, and APX, catalase (CAT), another crucial antioxidant enzyme, converts hydrogen peroxide into oxygen and water, rapidly lowering elevated ROS levels. These enzymes are particularly active in cell organelles like peroxisomes, and mitochondria, where ROS are predominantly generated, minimizing cell damage (Sharma and Ahmad, 2014). In Chinese cabbage (*Brassica rapa* subsp. pekinensis), activation of SOD was shown to suppress ROS accumulation, thereby reducing cell damage and improving drought tolerance and productivity (Chen et al., 2022). Similarly, in tomato (*Solanum lycopersicum*), drought and heat stress significantly increased ROS levels, which were counteracted by the activation of SOD, POD, APX, and CAT. These antioxidant enzymes played a crucial role in maintaining ROS homeostasis and preventing oxidative damage, particularly in drought-tolerant varieties (Zhou et al., 2019a). Likewise, in soybean (*Glycine max*), drought-tolerant cultivars exhibit enhanced antioxidant enzyme activities, effectively mitigating ROS-induced oxidative stress. Under split-root drought conditions, soybean plants significantly increased the activities of SOD, CAT, APX, and POD, which correlated with reduced H₂O₂ and MDA levels (Iqbal et al., 2019). Notably, drought-tolerant cultivars exhibited stronger antioxidant responses, maintaining higher chlorophyll content and photosynthetic efficiency compared to susceptible cultivars (Iqbal et al., 2019).

**Figure 2:**
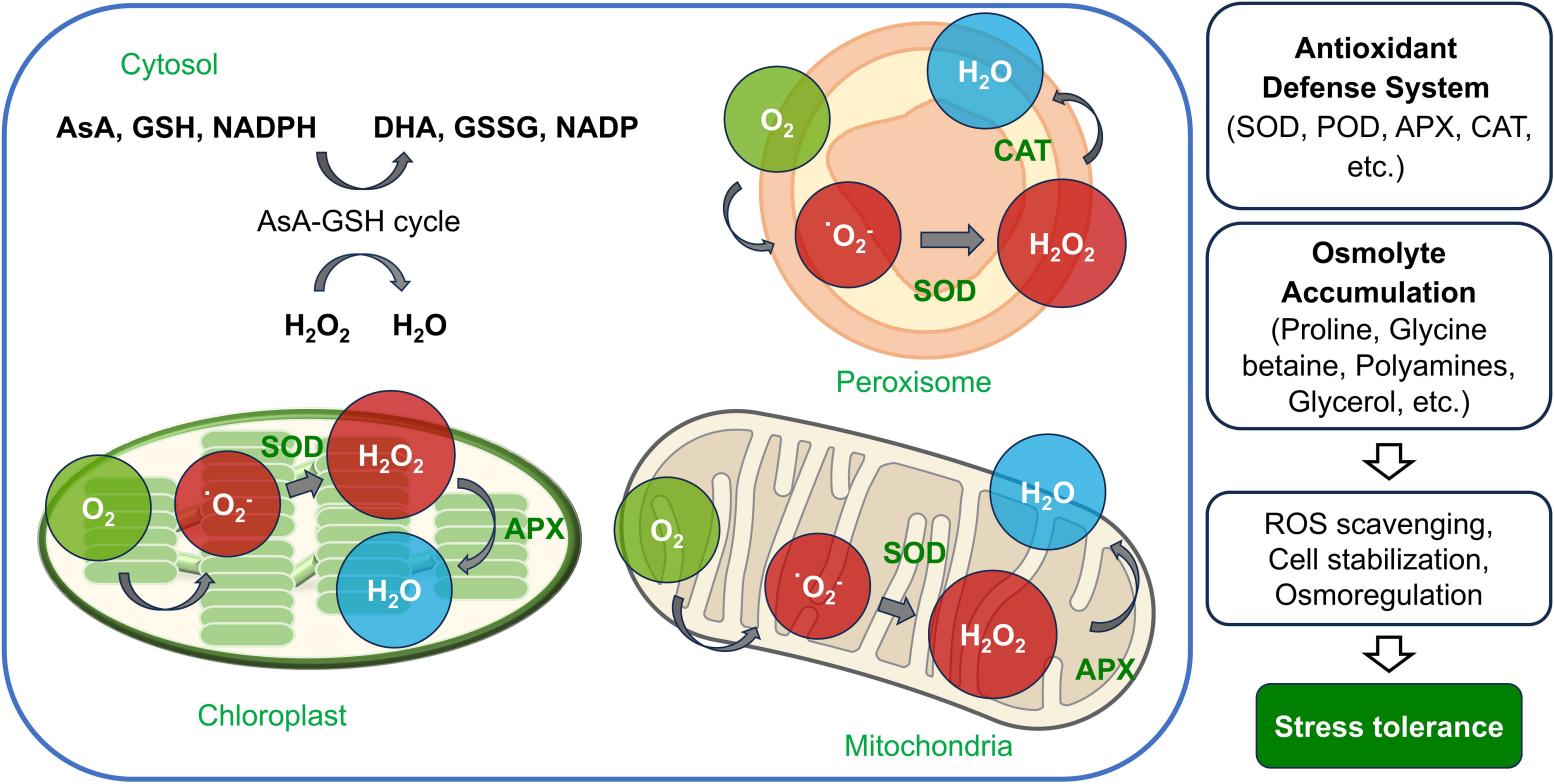
Antioxidant defense system in plant cell organelles under drought stress. The figure shows the generation and detoxification of reactive oxygen species (ROS) in chloroplasts, mitochondria, peroxisomes, and the cytosol. In chloroplasts and mitochondria, ROS such as superoxide ions (O₂⁻) are converted into hydrogen peroxide (H₂O₂) by superoxide dismutase (SOD). Ascorbate peroxidase (APX) further reduces H₂O₂ to H₂O, using ascorbate in the process. In peroxisomes, SOD also converts O₂⁻ to H₂O₂, which is then detoxified by catalase (CAT) to produce H₂O and oxygen (O₂). The cytosolic AsA-GSH cycle provides a continuous supply of antioxidants by recycling ascorbate and glutathione (GSH), helping to maintain cellular redox balance. Through the coordinated action of these enzymes, plants enhance stress tolerance, reducing oxidative damage in drought conditions.

Through the activation of these antioxidant enzymes, plants enhance their biochemical defense systems to mitigate environmental stresses and help maintain internal homeostasis (**Figure 2**).

### 4.2 Accumulation of osmolytes

Plants accumulate osmolytes as a biochemical mechanism to defend themselves against various stresses. Osmolytes are small organic compounds that help plants adapt to various abiotic stresses. These osmolytes include proline, glycine betaine, polyamines, glycerol, and mannitol, which play key roles in osmotic regulation, cell stabilization, and protein protection, allowing plant cells to maintain their function even under stress conditions (Chen and Jiang, 2010; Jogawat, 2019; Dikilitas et al., 2020). Proline accumulation occurs in response to a variety of stress conditions, such as drought, salinity, cold, and heavy metals, and has been reported to correlate with drought tolerance in several plant species (Iqbal et al., 2019; Mahmood et al., 2021; Shin et al., 2021; Plazas et al., 2022). Additionally, it scavenges ROS, mitigating the harmful effects of abiotic stresses (Hayat et al., 2012). In lettuce (*Lactuca sativa*), drought stress resulted in a significant accumulation of proline, with levels increasing approximately 660-fold on day 8 compared to control conditions. This dramatic increase suggests that proline plays a crucial role in osmotic adjustment and adaptation to drought stress by reducing oxidative damage and stabilizing cellular structures (Shin et al., 2021). In eggplant (*Solanum melongena*) and its wild relatives, proline accumulation increased significantly under drought stress; tolerant genotypes such as *S*. *incanum* and *S*. *pyracanthos* showed levels more than 79- and 95-fold higher, respectively than control plants, whereas susceptible genotypes showed only an 8-fold increase (Plazas et al., 2022). In drought-tolerant soybean (*Glycine max*) varieties, the accumulation of proline was significantly higher under drought stress in a split-root system compared to that in susceptible varieties (Iqbal et al., 2019). This increased concentration of proline is associated with enhanced water retention and osmotic regulation, both of which contribute to improved drought tolerance. However, recent findings indicate that the relationship between proline accumulation and drought tolerance may not be universally positive, as it can vary depending on the plant species and developmental stage. For example, in pepper (*Capsicum* spp.), bell pepper varieties such as Green Wonder exhibited higher proline levels under drought stress, but showed increased drought susceptibility rather than improved tolerance, suggesting a more complex interaction between osmotic regulation and stress adaptation (Mahmood et al., 2021). Glycine betaine, another important osmolyte, accumulates primarily in chloroplasts in response to drought stress and helps maintain photosynthetic efficiency through osmotic regulation (Gupta and Thind, 2015). Its primary functions include stabilizing proteins, regulating osmotic pressure, and scavenging ROS, thereby protecting plant cells and contributing to stress tolerance (Alasvandyari et al., 2017; Dikilitas et al., 2020). Despite the diverse biochemical/physiological effects of osmolytes on plants, further research is required for a deeper understanding of their impact on stress responses. Given their potential to mitigate the negative effects of ROS, osmolytes play an essential role in reducing agricultural losses caused by various environmental stresses (**Figure 2**).

## 5 Molecular biological response mechanisms

### 5.1 Hormonal regulation

**Figure 3:**
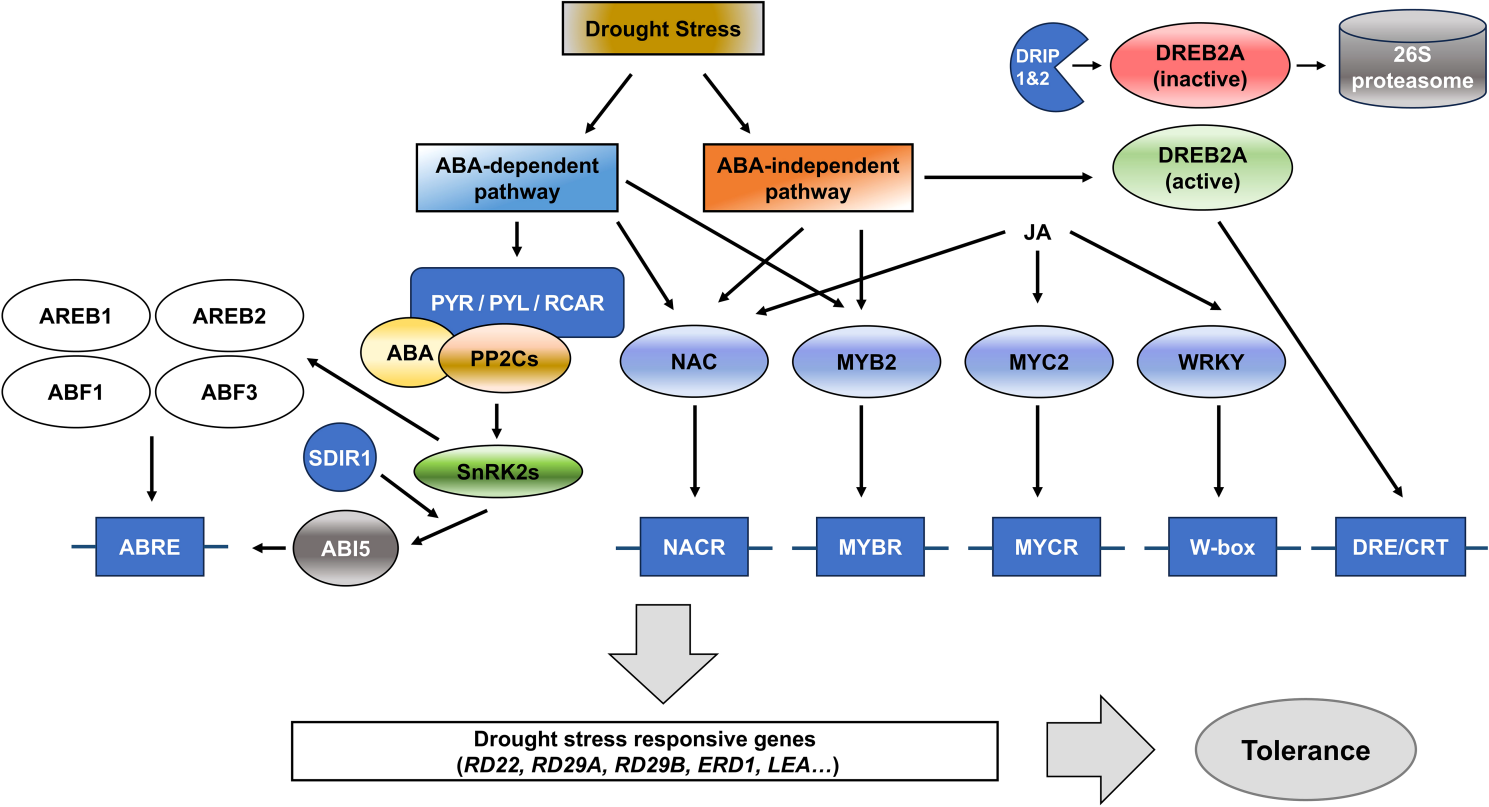
Schematic overview of ABA-dependent and ABA-independent signaling pathways involved in drought stress response. Upon drought stress, ABA is synthesized, leading to the activation of the ABA-dependent pathway, which includes ABA receptors (PYR/PYL/RCAR), PP2Cs, and SnRK2 kinases. This pathway regulates the expression of stress-responsive genes through transcription factors (TFs) like ABF/AREB families. In parallel, the ABA-independent pathway involves TFs such as NAC, MYB2, MYC2, and WRKY, which regulate the expression of drought-responsive genes such as RD22, RD29A, and ERD1 through binding to their respective regulatory elements (NACR, MYBR, MYCR, W-box, and DRE/CRT). The DREB2A TF is particularly important in the ABA-independent pathway and is negatively regulated by E3 ubiquitin ligases, DREB2A-INTERACTING PROTEIN1 (DRIP1)- and DRIP2. Together, these pathways enhance the plant tolerance to drought stress by modulating the expression of key stress-responsive genes.

Plants respond to various environmental stresses through hormonal regulation, controlling specific growth patterns and physiological changes. In drought stress environments, hormones such as abscisic acid (ABA), jasmonic acid (JA), salicylic acid (SA), and ethylene (ET) play complex roles in regulating stress responses (Abbas et al., 2023). ABA is a major hormone involved in abiotic stress response, influencing plant growth, germination, aging, and stress tolerance. ABA regulates drought stress responses by inducing stomatal closure through its signaling pathway and activating the expression of drought response genes (Yoon et al., 2020). ABA binds to multiple ABA receptors (PYR/PYL/RCARs), which inhibit the activity of PP2C, leading to the activation of SnRK2 and affecting downstream signaling pathways; the ABA receptors regulate the ABA signaling pathway by binding to ABA and inhibiting PP2C, thereby facilitating SnRK2 activation under stress conditions (Yin et al., 2009; Maszkowska et al., 2021) (**Figure 3**). On the other hand, gibberellin (GA), which regulates growth processes such as seed germination and flowering is suppressed under drought conditions. The deceleration of GA biosynthesis retards plant growth, thereby bolstering their chances of survival (Omena-Garcia et al., 2019). JA is primarily known for its role in defense against biotic stresses such as pests and pathogens, but it also plays a crucial role in abiotic stresses including drought, salinity, and heat (Ali and Baek, 2020). JA accumulates during drought stress and promotes stomatal closure to reduce water loss. It also activates antioxidant responses and regulates ion balance to minimize cell damage (Wang et al., 2020). SA contributes to drought tolerance by modulating ROS levels and regulating the expression of genes involved in stomatal regulation, often working in coordination with other hormones (Pandey and Chakraborty, 2015). ET, while primarily involved in fruit ripening and leaf aging also plays a crucial role in drought stress by balancing growth promotion and stress defense through stomatal regulation, aging acceleration, and stress-responsive gene expression (Chen et al., 2021a). These hormonal regulations are vital for plants to enhance their survival under extreme environmental conditions.

### 5.2 Drought stress response genes

Although plant hormones typically regulate similar physiological processes, each hormone functions through distinct transcription factors (TFs) or gene networks, ensuring non-redundant activity (Nemhauser et al., 2006). Under drought stress, many genes are regulated by ABA, highlighting its importance in the stress responses (Mizuno and Yamashino, 2008). Drought stress responses are generally divided into ABA-dependent and ABA-independent pathways (**Figure 3**). The TFs, AREB/ABFs (ABRE-binding protein/ABRE-binding factors) function within the ABA-dependent pathway by binding to the ABRE (ABA-responsive element) *cis*-acting element, while DREBs (DRE-/CRT-binding proteins) operate within the ABA-independent pathway by binding to the DRE/CRT (Dehydration-responsive element/C-repeat) cis-acting element present in the promoters of stress-responsive genes (Fujita et al., 2005; Yamaguchi-Shinozaki and Shinozaki, 2005; Yoshida et al., 2014). AREB1, AREB2, and ABF3 play critical roles in activating the expression of genes in the ABA-dependent pathway by binding to ABRE. When all of these TFs are functional, plants exhibit significantly enhanced drought tolerance (Yoshida et al., 2010). AREB/ABFs induce the expression of downstream genes such as *RD22* (*Responsive to Desiccation 22*) and *RD29B* (*Responsive to Desiccation 29B*), which are crucial for stress adaption by protecting cells and preserving intracellular water (Yamaguchi-Shinozaki and Shinozaki, 1993, 1994). Some LEA (Late Embryogenesis Abundant) genes contain ABRE sequences, which are bound by AREB/ABF transcription factors and increase the production of LEA proteins in response to drought stress. LEA proteins stabilize cell structures and protect critical molecules during cellular dehydration (Hundertmark and Hincha, 2008). Through the ABA-independent pathway, plants can react to drought stress without ABA signaling thanks to available DREB (Dehydration-Responsive Element-Binding) TFs that are a member of the plant-specific AP2/ERF (APETALA2/ethylene-responsive factor) family (Sakuma et al., 2002).

Specifically, DREB2A (Dehydration-Responsive Element-Binding protein 2A) controls the expression of genes that respond to drought stress, enhancing the capacity of plants to retain water (Sakuma et al., 2006). During the early phases of leaf turgor reduction, the *NCED3* (*9-cis-Epoxycarotenoid Dioxygenase*) gene, which is crucial for ABA biosynthesis, rapidly increases its expression, raising ABA levels under drought stress (Sussmilch et al., 2017). The *P5CS* (*Pyrroline-5-Carboxylate Synthetase*) gene is involved in proline biosynthesis, contributing to the antioxidant system of plants (Rai and Penna, 2013).

TF families such as NAC (NAM, ATAF1/2, and CUC2), MYB (myeloblastosis), and WRKY are also involved in controlling drought tolerance (**Figure 3**). The NAC TFs regulate downstream stress-responsive genes, helping plants adapt to drought and salinity (Nakashima et al., 2012); for example, overexpression of *NAC* gene family members including *ANAC019*, *ANAC055*, and *ANAC072* in *Arabidopsis* conferred drought tolerance (Tran et al., 2004). Similarly, transgenic plants overexpressing *AtMYC2* and/or *AtMYB2* showed higher sensitivity to ABA and the ABA-induced gene expression of *RD22* was enhanced in those transgenic plants. Furthermore, transgenic plants overexpressing both *AtMYC2* and *AtMYB2* displayed upregulated expression levels of several ABA-inducible genes, contributing to drought stress resistance (Abe et al., 2003). WRKY TFs, as plant-specific TFs are strongly and rapidly induced in response to certain abiotic stresses, such as drought and salinity. Moreover, it is known that they play crucial roles in both biotic and abiotic stress responses in plants (Seki et al., 2002). Overexpression of *GmWRKY54* in Arabidopsis and soybean (*Glycine max*) plants has been shown to enhance drought and salinity resistance through activating genes in ABA and Ca^2+^ signaling pathways (Zhou et al., 2008; Wei et al., 2019).

### 5.3 Protein stability and degradation

TFs and their downstream gene products are regulated through various plant hormones, inducing the stabilization or degradation of specific proteins to control intracellular protein levels in response to drought stress. Ubiquitin plays a critical role in protein stability and degradation, as well as in regulating the ABA signaling pathway during drought stress responses. The ubiquitin-proteasome pathway is a key mechanism for controlling intracellular protein degradation. Unnecessary or damaged proteins are tagged with ubiquitin and then degraded by proteasomes; this process is important for maintaining protein homeostasis within the cell, ensuring normal cellular functions by removing promptly damaged, misfolded or no longer needed protein (Moon et al., 2004; Sadanandom et al., 2012; Stone, 2019). Recent studies have highlighted the crucial role of the ubiquitin-proteasome pathway in improving crop yield and quality, particularly contributing to the regulation of seed germination rate, size, and nutrient composition (Varshney and Majee, 2022)

Ubiquitination is a three-step process involving three enzymes. The first enzyme, E1 (Ubiquitin-activating enzyme) uses ATP to activate ubiquitin. E2 (Ubiquitin-conjugating enzyme) transfers ubiquitin to E3 (Ubiquitin ligase), which recognizes target proteins and attaches ubiquitin, marking them for degradation by the 26S proteasome (Moon et al., 2004). The DREB2A TF that holds great importance in drought stress responsive gene expression is ubiquitinated and degraded by the E3 ligases DRIP1 (DREB2A-Interacting Protein 1) and DRIP2 under normal conditions. However, activities of DRIP1 and DRIP2 are suppressed under drought conditions, allowing DREB2A to activate downstream gene expression and enhance drought resistance (Qin et al., 2008).

In *Arabidopsis*, the RING-H2 zinc finger domain-containing E3 ligase XERICO (Greek for ‘drought tolerant’) has been shown to indirectly promote the expression of *NCED3*, a key gene in ABA biosynthesis, contributing to drought tolerance (Ko et al., 2006). Furthermore, ubiquitination can stabilize proteins, allowing them to persist longer in the cell and activate specific signaling pathways. For example, the SDIR1 (Salt- and Drought-Induced RING Finger 1) E3 ubiquitin ligase stabilizes the ABI5 (ABA-Insensitive 5) TF, enhancing drought resistance through the ABA signaling pathway (Zhang et al., 2007). The ubiquitin system is therefore crucial for raising crop quality and productivity, and it would be feasible to improve agricultural productivity and fortify tolerance to different environmental challenges by deeper understanding and utilizing the system.

## 6 Strategies for overcoming drought damage

### 6.1 Smart and traditional water management for drought resilience

Smart irrigation systems optimize water usage by monitoring soil moisture, weather, and plant needs, enabling precise watering regimes that shield vegetable crops from drought stress during cultivation. Recently, IoT (Internet of Things) technology has been employed to monitor soil moisture in real-time, enabling the implementation of automated irrigation systems. By adjusting water amounts based on the specific needs of crops, smart agriculture systems optimize crop health and yield (Bwambale et al., 2022). For instance, the pH and electrical conductivity (EC) of nutrient solutions are automatically adjusted in an IoT-based smart farming system while real-time climate conditions are monitored. This system increased the fresh weight (FW) of Chinese cabbage (*Brassica rapa* subsp. *pekinensis*) by 27.14% and dry weight (DW) by 48.9%, demonstrating the practical efficiency of smart agricultural systems in vegetable production (Promwee et al., 2024). In addition to real-time monitoring and automation, machine learning (ML) models have been increasingly integrated into smart irrigation systems to enhance decision-making processes. Recent studies have reported that Random Forest, a widely used ML algorithm, can effectively predict the yield of potato (*Solanum tuberosum*) and maize (*Zea mays*) based on rainfall and temperature data (Kuradusenge et al., 2023).

Furthermore, improving irrigation design is crucial for maximizing water use efficiency. A study on potato (*Solanum tuberosum*) demonstrated that optimizing drip irrigation systems can significantly improve both water use efficiency and crop productivity (Abdelraouf et al., 2020). Drip irrigation, which delivers water directly to the roots of plants, minimizes evaporation losses and ensures that crops receive adequate moisture, making it particularly effective in drought-prone environments (Abdelraouf et al., 2020). However, smart agriculture systems also face significant challenges. Many systems are limited to specific pests or crop types, restricting their broader applicability. Additionally, these systems often have high computational costs and dependencies on available data and hardware, which can lead to misclassification risks. Some systems tested on specific crops may struggle to generalize to different crops or agricultural environments (Jha et al., 2019). Traditional rainwater harvesting (RWH) practices can significantly increase crop productivity when combined with organic soil amendments. A recent study showed that the integration of *in-situ* RWH techniques, such as stone bunds and rainwater harvesting ponds, with poultry litter biochar increased the yields of maize (*Zea mays*) and barley (*Hordeum vulgare*) by 74% and 89.6%, respectively, while stand-alone RWH applications increased corn and barley yields by 6.7% and 36.2%, respectively. (Debebe et al., 2025). Integrating traditional water management techniques with smart irrigation systems could further enhance water efficiency in precision agriculture.

### 6.2 Biotechnology applications

A prominent solution to overcome drought damage in vegetable crops is the development of drought-resistant varieties through gene editing and/or breeding. In addition, transgenic plants with manipulated genes for gain/loss-of-function have been utilized to examine abiotic stress tolerance (Zhou et al., 2019b; Chen et al., 2021b; He et al., 2021; Li et al., 2021; Wang et al., 2022a, b; Yang et al., 2022a). Responsible genes for the stress-resistance traits are being introduced into other crops for various research purposes. Antisense RNA approaches for suppressing or regulating the expression of specific genes by forming double stranded RNA, disrupting translation and inhibiting the function of target genes have been also applied for generation of stress resistant crops (Deleavey and Damha, 2012). RNA interference (RNAi) techniques use short interfering RNA (siRNA) or short hairpin RNA (shRNA) for binding to target mRNAs and induce degradation, effectively silencing specific genes (Dykxhoorn and Lieberman, 2005). CRISPR/Cas9 is an RNA-guided endonuclease that specifically targets DNA sequences via nucleotide base pairing, resulting in permanent gene modifications (Hsu et al., 2014). The following table summarizes results from studies on transgenic plants with modified expression of specific genes related to drought stress: Developing stress-resistant crops based on the introduction of genes involved in stress responses is a rapid way to improve crop varieties (**Table 1**). For instance, the overexpression of a thaumatin-like protein gene, *BolTLP1* in broccoli (*Brassica oleracea* L. var. *Italica*) has been shown to improve both drought and salt tolerance (He et al., 2021). Similarly, transgenic *Arabidopsis* plants that overexpress the SR (serine/arginine-rich) protein gene, *BrSR45a* (also known as *BrSR-like 3*) and the DEAD-box RNA helicase gene *BrRH37* from cabbage (*Brassica rapa*) demonstrated improved drought resistance (Muthusamy et al., 2020; Nawaz et al., 2021). Ectopic expression of the β-carotene hydroxylase gene, *DcBCH1* from carrot (*Daucus carota* L.) resulted in enhanced carotenoid biosynthesis, contributing to drought tolerance, while the phytochrome-interacting factor gene, *DcPIF3* of carrot was found to be associated with ABA-related drought responses (Li et al., 2021; Wang et al., 2022b). Additionally, studies in chickpea (*Cicer arietinum* L.) have revealed that the overexpression of a glutaredoxin gene, *CaGrx*, contributes to ROS scavenging and improves drought tolerance (Kumar et al., 2023). In cucumber (*Cucumis sativus* L.), overexpression of *CsATAF1*, which encodes a NAC transcription factor activated ABA-dependent signaling pathways, resulting in enhanced drought tolerance (Wang et al., 2018). CRISPR/Cas9 genome editing systems have also been applied to improve drought resistance in tomato (*Solanum lycopersicum*); knockout of the pathogenesis-related gene 1, *SlNPR1* resulted in increased stomatal opening, leading to increased sensitivity to drought (Li et al., 2019). Similarly, knockout mutants of the auxin response factor gene, *SlARF4* exhibited drought tolerance, with increased expression levels of genes in the phenylpropanoid biosynthetic pathway, potentially contributing to lignin biosynthesis, vascular development, and enhanced drought resistance (Chen et al., 2021b).

**Table 1:** Drought stress study in transgenic plants with regulated gene expression.

Crop	Editing	Gene	Changes under drought stress	Reference
Broccoli (*Brassica oleracea* L. var. *Italica*)	Overexpression	*BolTLP1*	Drought and salt tolerance	He et al., 2021
Cabbage (*Brassica rapa*)	Overexpression in *Arabidopsis*	*BrSR45a*	Drought tolerance	Muthusamy et al., 2020
		*BrRH37*	Drought tolerance	Nawaz et al., 2021
Carrot (*Daucus carota* L.)	Overexpression in *Arabidopsis*	*DcBCH1*	Increased ABA levels through carotenoid biosynthesis, drought tolerance	Li et al., 2021
		*DcPIF3*	ABA related and drought tolerance	Wang et al., 2022b
Chickpea (*Cicer arietinum* L.)	Overexpression in *Arabidopsis*	*CaGrx*	ROS scavenging, drought tolerance	Kumar et al., 2023
Cucumber (*Cucumis sativus* L.)	Overexpression	*CsATAF1*	ABA-dependent pathway, ROS scavenging, drought tolerance	Wang et al., 2018
	Overexpression in *Nicotiana tabacum*	*CsPLDα*	Increased stomatal closing, drought tolerance	Ji et al., 2018
Eggplant (*Solanum melongena* L.)	Overexpression in *Arabidopsis*	*SmAPX*	Enhanced APX activity, Drought tolerance	Chiang et al., 2017
Konjac (*Amorphophallus konjac*)	Overexpression in *Arabidopsis*	*AkCSLA*	Drought sensitivity	Luo et al., 2024
Melon (*Cucumis melo* L.)	Overexpression in *Arabidopsis*	*CmXTH11*	Cell wall flexibility, drought tolerance	Zhao et al., 2024
Oriental melon (*Cucumis melo* var. *makuwa* Makino)	Overexpression in *Arabidopsis*	*CmLOX10*	JA signaling mediated, Drought tolerance	Xing et al., 2020
Pepper (*Capsicum annuum* L.)	Overexpression in *Arabidopsis*	*CaNAC46*	Drought and salt tolerance	Ma et al., 2021
		*CaDHN3*	ROS scavenging, drought and salt tolerance	Meng et al., 2021
Potato (*Solanum tuberosum* L.)	Overexpression in *Arabidopsis*	*StNAC053*	Drought and salt tolerance	Wang et al., 2021
	Overexpression in *Gossypium barbadense* L	*StDREB2*	ROS scavenging, drought tolerance	El-Esawi and Alayafi, 2019
Pumpkin (*Cucurbita moschata*)	Overexpression in *Arabidopsis*	*CmNAC1*	Drought, salt and cold tolerance	Cao et al., 2017
Soybean (*Glycine max* (L.) Merr.)	Overexpression	*GmWRKY54*	ABA signalling pathway, drought tolerance	Wei et al., 2019
	Overexpression	*GmERF144*	Drought tolerance	Wang et al., 2022a
	Overexpression	*AtNCED3*	Drought tolerance, reduced yield loss	Molinari et al., 2020
	Overexpression in *Arabidopsis*	*GmMYB118*	Activated stress-responsive genes, drought and salt tolerance	Du et al., 2018
	Overexpression & CRISPR/Cas9 (knock-out)	*GmNAC12*	Drought tolerance	Yang et al., 2022a
Strawberry (*Fragaria* × *ananassa* Duch.)	Overexpression in *Arabidopsis*	*FaTEDT1L*	Drought tolerance	Chu et al., 2024
Sweet potato (*Ipomoea batatas* [L.] Lam)	Overexpression	*IbCBF3*	Drought and cold tolerance	Jin et al., 2017
	Overexpression & RNAi	*IbMYC2*	Drought and salt tolerance	Hu et al., 2024
	Overexpression in *Arabidopsis*	*IbMYB116*	JA signaling pathway, ROS scavenging, drought tolerance	Zhou et al., 2019b
		*IbNAC3*	Drought and salt tolerance	Meng et al., 2023
Tomato (*Solanum lycopersicum* L.)	Overexpression	*SlGATA17*	Phenylpropanoid biosynthesis pathway, drought tolerance	Zhao et al., 2021
	Overexpression in *Arabidopsis*	*SlERF5*	Drought and salt tolerance	Pan et al., 2012
	CRISPR/Cas9 (knock-out)	*SlNPR1*	Increased stomatal opening, drought sensitivity	Li et al., 2019
	CRISPR/Cas9 (knock-out)	*SlARF4*	Phenylpropanoid biosynthesis pathway, drought tolerance	Chen et al., 2021b
	RNAi	*SlNAC11*	Drought sensitivity	Wang et al., 2017

However, since this process relies on the expression of specific stress-related genes, it has limitations. It is important to not only develop transgenic crop varieties but also integrate methods that enhance gene expression or utilize antioxidant enzymes. Also, it is still necessary to evaluate transgenic plants under stress conditions and understanding the physiological impacts of introduced/modified genes at the whole-plant level.

Recent research has increasingly focused on enhancing drought tolerance through NPBTs with conventional breeding approaches to generate resilient crop types (Shao et al., 2018). These activities are crucial for supporting food security and promoting sustainable agricultural development. The development of GMOs, particularly gene-edited crops, offers new prospects in agriculture, significantly aiding efforts to counteract declines in agricultural productivity due to climate change (Qaim, 2020). To maximize the benefits of such technologies, it is essential to take into account the regulatory framework governing GMOs and/or gene editing products. Policies regarding gene-edited crops differ significantly across the globe; certain countries implement more permissive regulations that facilitate the commercial cultivation and research of these crops, whereas others uphold stringent regulations that restrict their development and commercialization (Wolt et al., 2016). These international regulatory differences significantly influence the global distribution and adoption of GMOs and/or gene-edited products.

Moreover, the public’s understanding and perception of gene editing and GMO technologies demonstrate considerable regional disparities, which affect the acceptance and application of these technologies. This underscores the importance of transparent communication and education concerning the benefits and scientific safety of gene editing, with the aim of enhancing public awareness and fostering responsible utilization. Identifying target genes for gene editing approaches through genomics and gene function studies is also essential. By understanding the roles of specific genes in stress responses, gene editing technologies can be applied with greater precision and effectiveness. The ongoing exploration and evaluation of potential target genes are vital steps in generation of climate-resilient crops with improved levels of abiotic stress resistance. From a policy perspective, it is essential to establish equitable and science-based regulatory frameworks to mitigate adverse effects and ensure the responsible integration of these technologies into agricultural systems.

### 6.3 Grafting and genomics-assisted breeding for drought tolerance

A variety of non-GM approaches have been used to improve drought tolerance in vegetable crops (Zhang et al., 2019; Shehata et al., 2022). Unlike genetic modification techniques that introduce foreign DNA, these methods rely on conventional breeding, marker-assisted selection (MAS), and physiological modifications through grafting (Kumar et al., 2017; Liu et al., 2023a). One of the most widely used methods is grafting, which has been shown to improve drought resistance by increasing water uptake efficiency and activating stress-responsive mechanisms. In cucumber (*Cucumis sativus*), grafting onto drought-tolerant rootstocks significantly increased the activity of antioxidant enzymes and promoted the accumulation of osmoprotectants such as proline. Additionally, the grafted plants exhibited up-regulated expression of stress-responsive genes, such as *CsAGO1* and *CsDCLs*, which contributed to improved drought tolerance (Shehata et al., 2022). In tomato (*Solanum lycopersicum*), grafting improved photosynthetic capacity, maintained chlorophyll content, and increased antioxidant enzyme activities, resulting in reduced oxidative stress and improved drought tolerance. Additionally, the grafted plants exhibited better stomatal regulation and higher water use efficiency under drought conditions (Zhang et al., 2019). In watermelon (*Citrullus lanatus*), grafting onto drought-tolerant rootstocks such as wild watermelon (*Citrullus colocynthis*) significantly increased antioxidant enzyme activities, reduced oxidative damage, and improved plant water status under drought conditions (Shehata et al., 2022).

Despite these advantages, however, grafting has several limitations and challenges. Grafting is a labor-intensive process that requires skilled personnel and post-graft management, making it costly and difficult for large-scale production. These factors are significant barriers to the widespread adoption of grafting, especially for smallholders with limited resources (Rasool et al., 2020; Lee et al., 2010). Additionally, successful grafting requires physiological and genetic compatibility between the rootstock and the scion. In some combinations, the accumulation of phenolic compounds at the graft interface inhibits vascular differentiation, leading to impaired water and nutrient transport and, ultimately, graft failure (Goldschmidt, 2014; Pina et al., 2012). Although grafting improves drought tolerance, its effectiveness may be limited under extreme drought conditions. Therefore, for long-term drought mitigation, grafting should be combined with other agronomic strategies, such as mulching and the application of biostimulants, to ensure optimal plant growth and productivity (Pina et al., 2017).

Recent advancements in single nucleotide polymorphism (SNP)-based MAS have significantly improved the accuracy of selecting drought-tolerant crop varieties. SNPs are variations in a single nucleotide at a specific position in the genome, enabling the precise identification of drought-tolerant genotypes (Nassar et al., 2018; Zhou et al., 2023). Compared to conventional breeding, MAS enables direct identification of drought-associated genotypes through genome-wide association studies (GWAS), accelerating the development of drought-resistant varieties (Liu et al., 2023a). Moreover, to improve the efficiency of selecting drought-tolerant vegetable crops, GWAS have been extensively utilized to identify quantitative trait loci (QTLs) linked to drought adaptation across various species. For example, a GWAS conducted on chickpea (*Cicer arietinum*) identified 1,344 SNP markers, of which 22 were significantly associated with drought tolerance across different environments (Istanbuli et al., 2024). Similarly, QTL analysis in *Brassica napus* identified loci associated with root morphology and water-use efficiency, which are essential traits for drought adaptation (Jayarathna et al., 2024). Beyond these species, GWAS have also been employed in potato (*Solanum tuberosum*) to identify QTLs involved in drought response at the tuber initiation stage, leading to the discovery of 38 significant genomic regions associated with physiological, biochemical, and yield-related traits under water deficit conditions (Díaz et al., 2021). These studies demonstrate that combining MAS with QTL mapping can enhance the efficiency of selecting drought-tolerant genotypes, thereby supporting the application of genome-based breeding approaches for crop improvement.

### 6.4 Use of various biostimulants

**Figure 4:**
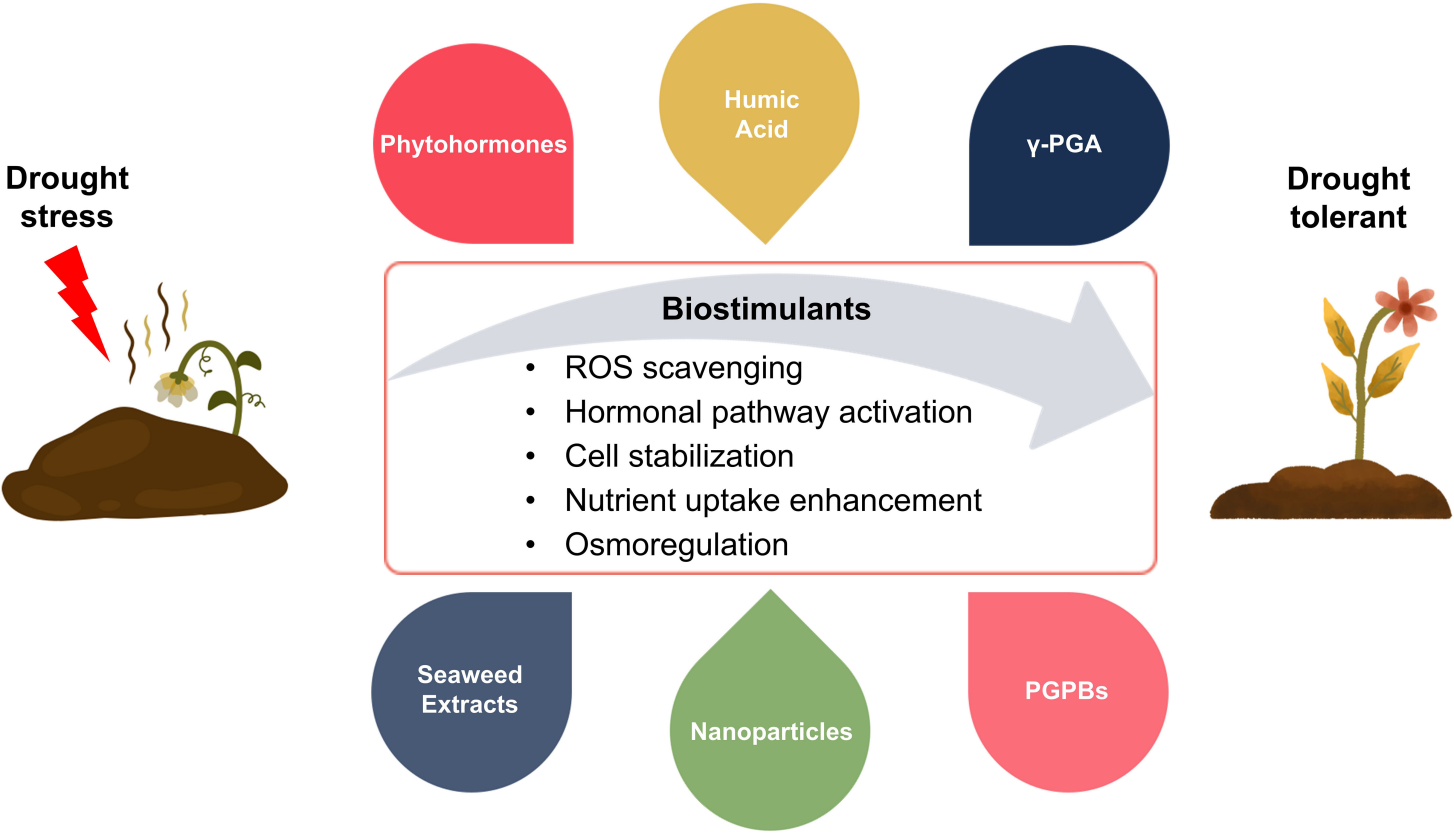
Figure illustrates the mechanisms and types of biostimulants used to enhance drought stress tolerance in vegetable crops. Biostimulants, including phytohormones, humic acid, γ-glutamic acid (γ-PGA), seaweed extracts, nanoparticles, and plant growth-promoting bacteria (PGPBs), improve plant tolerance under drought conditions. Biostimulants strengthen drought resistance by promoting ROS scavenging, activating hormonal pathways, stabilizing cellular structures, improving nutrient uptake, and regulating osmotic balance, thereby increasing plant growth, productivity, and stress resistance.

Another strategy to reduce drought damage to vegetable crops is the use of biostimulants (**Figure 4**). The market of plant biostimulants is expanding, and they are being regarded as innovative agricultural tools (Povero et al., 2016). Agricultural biostimulants can be used as substitutes for synthetic chemical pesticides like insecticides and herbicides, helping to make agriculture more resilient and sustainable (Bulgari et al., 2015; Van Oosten et al., 2017). Plant biostimulants, which can improve plant growth and production under abiotic stress conditions are made from a variety of organic and inorganic materials as well as microorganisms (Du Jardin, 2015; Goñi et al., 2018). By improving soil conditions, biostimulants have a direct impact on plant physiology and metabolism; increasing the efficiency of water and nutrient usage, promoting plant growth, and enhancing primary and secondary metabolism to assist plants cope with abiotic stress (Bulgari et al., 2015).

Humic substances (HSs) such as humic acid, fulvic acid, and humin are naturally occurring components of soil organic matter and have been shown to enhance drought resistance when applied to plants (Trevisan et al., 2010). For example, when humic acid-based biostimulants were applied to lettuce (*Lactuca sativa*), the plants showed improved nutrient absorption capacity, leading to enhanced quality and increased resistance to abiotic stress (Savarese et al., 2022). In melon (*Cucumis melo* L.), the application of humic acid increased the accumulation of potassium (K) and calcium (Ca) ions, chlorophyll content, and the activity of antioxidant enzymes like SOD and CAT, thereby improving drought resistance (Kıran et al., 2019). As soil conditioners and biostimulants, seaweed extracts were also utilized in agriculture because they included compounds that functioned similarly to plant hormones to stimulate plant growth (Battacharyya et al., 2015). Applying seaweed extracts to broccoli (*Brassica oleracea* var. *cymosa* L.) enhanced resilience to abiotic stresses and improved production and quality (Gajc-Wolska et al., 2012). These results suggest that seaweed extracts can be useful for enhancing the productivity of high-value crops such as broccoli. Microorganisms also play roles in improving soil health and promoting plant growth. PGPB (Plant Growth Promoting Bacteria) enhance the production of osmolytes and regulate hormonal pathways in plants. They also help plants withstand abiotic stresses such as heat, salinity, and drought by improving root development and water uptake (Turan et al., 2017). Similarly, γ-glutamic acid (γ-PGA), a biodegradable and non-toxic polymer produced by microorganisms, has attracted attention for its potential applications in agriculture. γ-PGA improves nitrogen absorption under soil conditions, enhances water retention capacity in *Brassica napus* L., and promotes the removal of ROS while facilitating the accumulation of the osmoprotectant proline under drought stress (Xu et al., 2020). Biostimulants not only promote plant growth but also modulate stress signaling and regulate gene transcription, thereby fundamentally strengthening crop resilience. In particular, γ-PGA regulates plant stress response pathways by activating the expression of ABA biosynthesis-related genes and contributes to enhancing the activity of antioxidant enzymes such as SOD, CAT, APX, and POD (Xu et al., 2020). Thus, the mechanisms of various biostimulants are involved in enhancing cellular and metabolic responses, such as stress signaling, ROS scavenging, and transcriptional activation. Nanoparticles have recently attracted more attention from researchers due to their functional roles that are comparable to those of biostimulants. The application of proper concentration of titanium dioxide (TiO₂) and zinc oxide (ZnO) nanoparticles was shown to promote photosynthesis and nutrient absorption in tomato (*Solanum lycopersicum* L.), demonstrating the potential to enhance abiotic stress tolerance. However, high concentrations of nanoparticles can cause physiological disorders and inhibit growth, so further research is needed to establish optimal concentrations for application across various crop species (Raliya et al., 2015). Another approach to confer drought resistance is the application of exogenous hormones or bioactive substances, which can induce positive changes in crops under stress conditions (**Table 2**).

**Table 2:** Effects of exogenous hormone and bioactive substance treatments on vegetable crop responses under drought stress.

Biostimulant/Exogenous Application	Crops	Effects of crops under drought stress	Reference
Abscisic Acid	Lettuce, Sweet potato	Increased sugar content, antioxidant enzyme activity, improved drought tolerance	Urbutis et al., 2024; Zhang et al., 2020
Ascorbic Acid	Pepper, Sweet pepper	Improved antioxidant defense, drought tolerance, enhanced physiological responses	Khazaei and Estaji, 2020; Khazaei et al., 2020
Benzoic Acid	Soybean	Improved gas-exchange and chlorophyll contents	Anjum et al., 2013
Brassinosteroid	Chinese cabbage	Improved osmotic regulation, antioxidant defense	Ahmad Lone et al., 2022
γ-glutamic acid	Rapeseed	Enhanced antioxidant enzyme activity, activated ABA biosynthesis	Xu et al., 2020
L-glutamic acid	Tomato	Increased proline content, reduced ROS accumulation, enhanced antioxidant enzyme activity	Kim and Kwak, 2023
Humic Acid	Lettuce, Melon	Improved nutrient absorption, increased K and Ca ion accumulation, improved nutrient absorption capacity, enhanced drought resistance	Savarese et al., 2022; Kıran et al., 2019
Hydrogen Peroxide	Cucumber	Improved drought tolerance	Sun et al., 2016
Jasmonic Acid	Chinese cabbage, Strawberry	Improved osmotic regulation, antioxidant defense	Ahmad Lone et al., 2022; Yosefi et al., 2020
Kinetin	Lettuce	Enhanced productivity, improved drought tolerance	Urbutis et al., 2024
Melatonin	Pea	Improved antioxidant defense, growth and photosynthetic efficiency	Ahmad et al., 2023
PGPB (Plant Growth Promoting Bacteria)	Broccoli	Improved growth, yield, antioxidant enzyme activity	Kim et al., 2020
	Common bean	Improved root growth, water absorption, osmolyte synthesis, and drought tolerance	Figueiredo et al., 2008
	Pea	Improved growth, yield, antioxidant enzyme activity	Arafa et al., 2021
Protein Hydrolysate-Based Biostimulant	Paper, Tomato	Enhanced root growth, improved shoot biomass, increased water-use efficiency, and elevated antioxidant enzyme activity	Agliassa et al., 2021; Francesca et al., 2021
Salicylic Acid	Chinese cabbage, Cucumber, Sweet potato	Improved drought tolerance, enhanced growth and yield	Cha et al., 2020; Baninasab, 2010; Huang et al., 2022
Seaweed Extracts	Broccoli, Tomato	Improved drought tolerance, production and quality	Gajc-Wolska et al., 2012; Kałużewicz et al., 2017; Goñi et al., 2018
Serotonin	Tomato	Increased antioxidant activity	Akcay and Okudan, 2023
Zinc Oxide Nanoparticles	Eggplant, Melon	Improved photosynthetic efficiency, growth, yield, and antioxidant defense	Semida et al., 2021; Rehman et al., 2023

## 7 Conclusions

The sustainable production of vegetable crops, which is essential for ensuring global food security, is seriously threatened by the increasingly extreme weather patterns brought on by climate change. Among the many impacts of climate change, drought stress, interferes with physiological, biochemical, and molecular mechanisms of plants from germination to maturation, resulting in decreased productivity.

For key vegetable crops, drought stress has been shown to cause leaf senescence and reduce photosynthetic efficiency. In response to drought-induced ROS, the activity of antioxidant enzymes such as SOD, POD, CAT, and APX was increased. These enzymes contributed to reducing ROS accumulation and minimizing cellular damage, thereby enhancing crop survival. Through the ABA signaling pathway, stomatal closure and the activation of drought-responsive gene expression were triggered, while other hormones like GA and JA also induced various physiological changes, increasing plant tolerance to drought.

The use of climate smart agricultural systems and biostimulants positively influence the growth and productivity of vegetable crops. Smart irrigation systems, in particular, optimized crop health and yield by monitoring soil moisture in real time. Additionally, various biostimulants proved effective in enhancing drought resistance in crops such as lettuce and melons, showing potential for increasing tolerance to abiotic stresses. However, further research is necessary to determine the optimal concentrations for the application of nanoparticles, as high concentrations have been shown to induce stress and inhibit growth.

Future research should further elucidate the complex interactions among transcription factors, hormone signaling pathways, and drought-responsive genes. While gene editing technologies hold great promise, long-term evaluations are necessary to ensure their stability, efficiency, and regulatory viability across various crop species. Similarly, optimizing biostimulant applications, nanoparticle utilization, and smart irrigation systems will be crucial for maximizing their impact under drought conditions. An integrated approach that combines gene editing, molecular breeding, smart irrigation, and biostimulant applications will be essential for climate-resilient vegetable cultivation and production, thereby ensuring long-term global food security.

## Glossary

ABA: Abscisic acid
JA: Jasmonic acid
ET: Ethylene
ROS: Reactive oxygen species
NPBT: New plant breeding technologies
GMO: Genetically modified organisms
SOD: Superoxide dismutase
POD: Peroxidase
APX: Ascorbate peroxidase
CAT: Catalase
PP2C: Protein Phosphatase 2C
SnRK2: Sucrose Non-Fermenting Related Kinase 2
GA: Gibberellin
AREB/ABFs: ABRE-binding protein/ABRE-binding factors
ABRE: ABA-responsive element
DRE/CRT: Dehydration-responsive element/C-repeat
*RD22*: *Responsive to Desiccation 22*
*RD29B*: *Responsive to Desiccation 29B;* LEA, Late Embryogenesis Abundant
DREB: Dehydration-Responsive Element-Binding
AP2/ERF: APETALA2/ethylene-responsive factor
DREB2A: Dehydration-Responsive Element-Binding protein 2A
*NCED3*: *9-cis-Epoxycarotenoid Dioxygenase*
*P5CS*: *Pyrroline-5-Carboxylate Synthetase;* NAC, NAM, ATAF1/2, and CUC2
MYB: myeloblastosis
E1: Ubiquitin-activating enzyme
E2: Ubiquitin-conjugating enzyme
E3: Ubiquitin ligase
DRIP1: DREB2A-Interacting Protein 1
ABI5: ABA-Insensitive 5
SDIR1: Salt- and Drought-Induced RING Finger 1
TF: Transcription factor
PGPB: Plant Growth Promoting Bacteria
SNP: Single nucleotide polymorphism
GWAS: Genome-wide association studies
QTL: Quantitative trait loci.
